# Protective practices against tick bites in Denmark, Norway and Sweden: a questionnaire-based study

**DOI:** 10.1186/s12889-019-7613-4

**Published:** 2019-10-22

**Authors:** Martin Tugwell Jepsen, Pikka Jokelainen, Solveig Jore, Anders Boman, Daniel Slunge, Karen Angeliki Krogfelt

**Affiliations:** 1 Department of Bacteria, Parasites and Fungi, Statens Serum Institut, Copenhagen, Denmark; 2 Department of Virus and Microbiology Special Diagnostics, Statens Serum Institut, Copenhagen, Denmark; 3 Norwegian Institute of Public Health, Oslo, Norway; 4 Department of Economics, University of Gothenburg, Gothenburg, Sweden; 5 Gothenburg Centre for Sustainable Development, GMV, University of Gothenburg, Gothenburg, Sweden; 6 Department of Science and Environment, Roskilde University, Roskilde, Denmark

**Keywords:** Scandinavia, Nordic countries, Europe, Tick, Protective behaviour

## Abstract

**Background:**

Tick-borne infections are of emerging and increasing concern in the Scandinavian countries Denmark, Norway and Sweden. Only few studies have investigated protective practices against tick bites in the general population. The aim of this multi-country study was to assess the use of protective practices and the perception of the efficacy of them.

**Methods:**

We surveyed the extent of using protective practices against tick bites, using the same questionnaire in three local languages. In addition, we surveyed perceptions of how good a protection the different practices provide. Altogether 783 individuals from Denmark, 789 from Norway and 1096 from Sweden participated in the study by completing an extensive online questionnaire in October 2016.

**Results:**

Altogether 1011 respondents (37.9%) reported using at least three different protective practices either often or always when in areas where there are ticks, while 522 (19.6%) reported using none. Female gender was among the factors identified as positively associated with using several of the specific practices often or always when in areas where there are ticks. The gender-difference in extent of using protective practices against tick bites was particularly pronounced in Sweden. Based on a multivariable logistic regression model, being female, being from Sweden, and having experienced one or more tick bites were positively associated with using at least three different protective practices against tick bites either often or always when in areas where there are ticks (odds ratios 1.90, 1.87 and 1.88, respectively).

**Conclusions:**

The results of our study, especially the observed differences by country and by gender, can be useful in targeting future information to the public. In particular, our results suggest that men across all ages should be considered a specific target group for this information.

## Background

Ticks, especially *Ixodes ricinus*, have been expanding northwards in latitude and to higher altitudes in Europe, as well as in the three Scandinavian countries Denmark, Norway and Sweden [1, 2]. As a result, tick-borne infections are of increasing public health concern. Of the human tick-borne diseases, Lyme borreliosis is the most common, and several endemic foci of tick-borne encephalitis (TBE) have been identified in the region [3–5].

There are reports on protective practices against tick bites from endemic regions [5–10] but relatively little is known about protective practices against tick bites in the Scandinavian countries. Two studies from Sweden were identified, one studied protective practices against tick bites in a highly endemic area [11] and another was a nationwide survey [12]. Literature searches found no published studies on protective practices against tick bites in Denmark and Norway. Improved knowledge about the different protective practices used against tick bites is hence needed to develop public health strategies that can prevent tick-borne infections.

The aims of this study were to survey the protective practices against tick bites as well as the perception of the protection they provide in Scandinavia. Previous studies have shown that the use of protective practices is highly dependent on the epidemiological status and risk context [6, 12]. Through describing and comparing protective practices among the general public in Denmark, Norway and Sweden, this study provides new knowledge which we believe is a good basis for strategical, targeted measures for preventing tick-borne diseases.

## Methods

### Setting and study design

As part of a large-scale investigation into different aspects related to ticks and selected tick-borne infections in Denmark, Norway, and Sweden, we surveyed the protective practices against tick bites. The descriptive study was cross-sectional, multi-country and questionnaire-based.

**Fig. 1:**
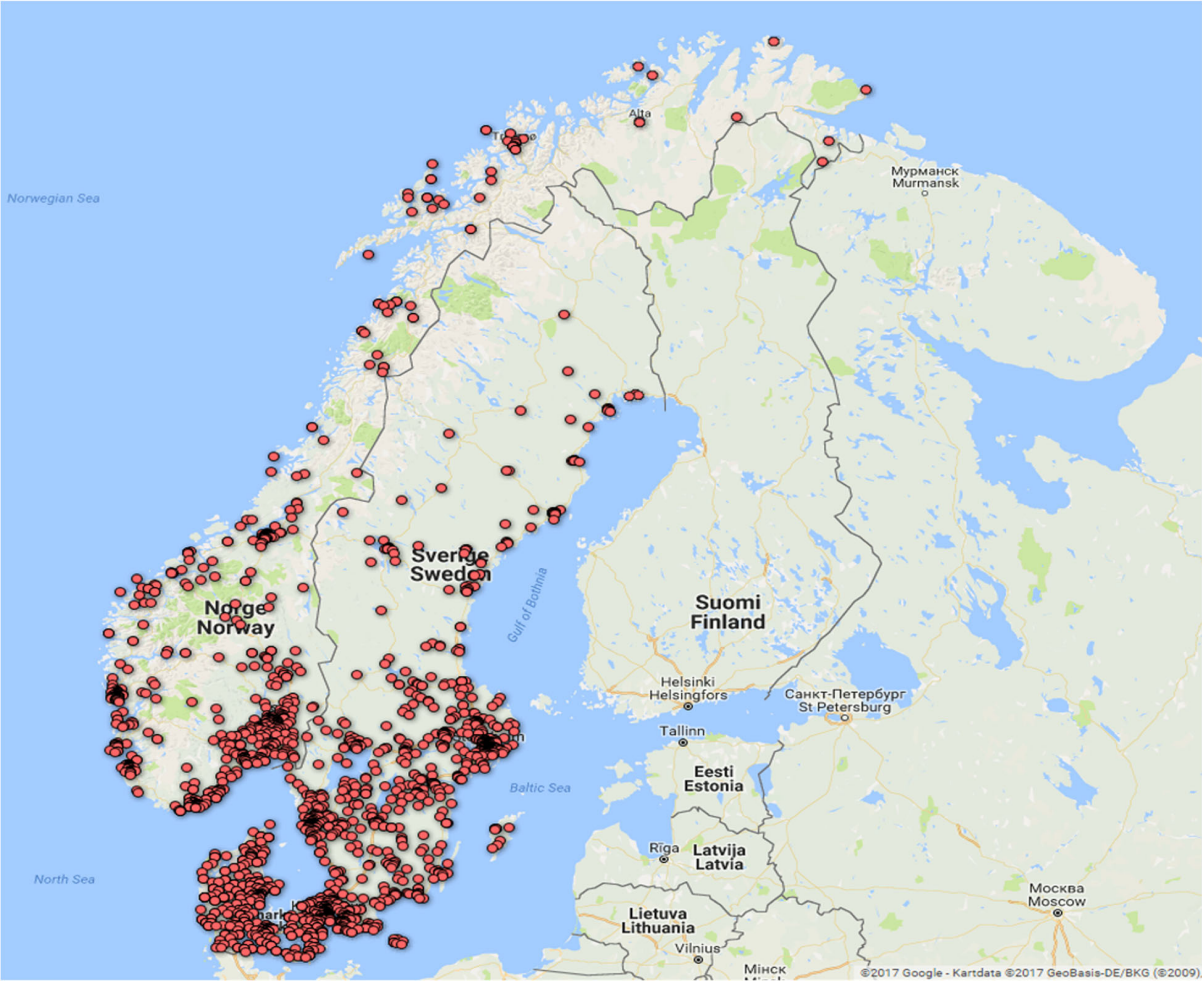
Residence of the respondents, each marked by a red dot. Map created by authors using Google Maps, 2017 [13]

An external analytics company (Epinion) facilitated the questionnaire part of the investigation. The respondents were randomly selected from national telephone registries within each country. Initially 22,191 individuals in the three Scandinavian countries were contacted by telephone and/or e-mail, and among these, 1436 individuals in Denmark, 1518 individuals in Norway, and 2037 individuals in Sweden expressed willingness to participate. A total of 783, 789 and 1096 individuals from the three countries, respectively, completed the questionnaire. The geographical distribution of the respondents is shown in Fig. 1 where each red dot represents one respondent.

### Questionnaire

A skip-pattern questionnaire was designed and translated into three languages (Danish, Norwegian, and Swedish). A pilot test of the questionnaire was performed in August 2016 and after minor adjustment of wording and use of scales, the full questionnaire was distributed in October 2016. Data collection was carried out by the analytics company (Epinion). The 44 questions covered demographic details and experiences, practices, knowledge and risk perception related to ticks and selected tick-borne diseases (Additional file 1).

### Inclusion of data and statistical analyses

The primary question analysed in this study was: “To what extent do you use the following practices to protect yourself against tick bites when in a forest or a field or other places where you may encounter ticks?” The six practices listed were wearing long trousers and long-sleeved clothes; using repellent; tucking trousers in socks; avoiding high grass and bushes; checking the body for ticks while outdoors; and checking the body for ticks after being outdoors. Moreover, those who had children were asked about checking the children for ticks, and those who had pets were asked about checking the pets for ticks. The options were “Never”, “Rarely”, “Often”, and “Always”. We also summarise the answers to the question: “How good a protection would you say each of the following practices provides against tick bites and tick-borne diseases?” The practices listed were the same as for the first question, except no question was asked about the protective effect on checking children. The options were “No protection”, “Rather poor protection”, “Quite good protection”, and “Very good protection”.

The statistical analyses focused on the reported extent of using protective practices and were performed using Stata 13.1 (StataCorp, College Station, TX, USA). For the statistical analyses, we dichotomised the extent of use of each of six protective practices (never or rarely vs. often or always) and used them as outcomes in the univariable (crude) analyses. An alternative outcome, one used also in a simple multivariable model, was ‘using at least three of the six practices often or always’, which was compared with using them never or rarely. This outcome was selected to illustrate active use of protective practices.

**Fig. 2:**
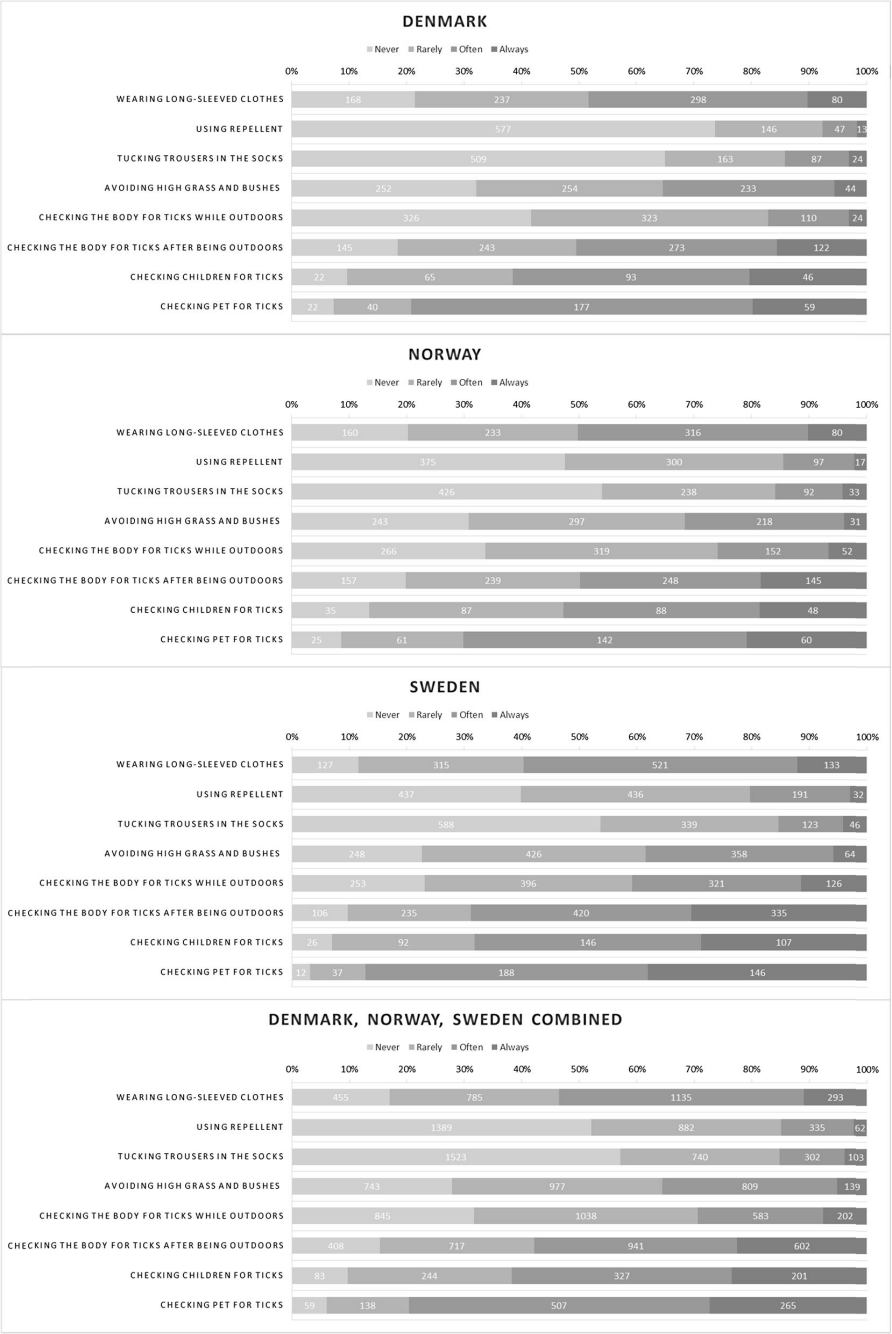
The number and proportion of respondents selecting each answer about protective practices against tick bites, by country and the three countries combined

**Fig. 3:**
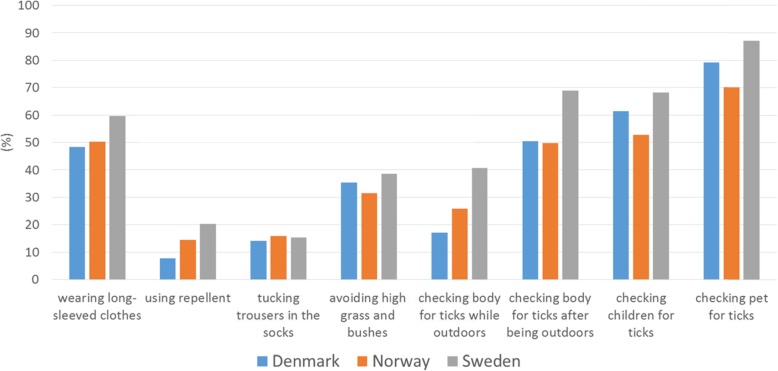
The combined proportion of respondents who reported ‘Often’ or ‘Always’ using each of the protective practices against tick bites, by country. The number of respondents varied by question

**Fig. 4:**
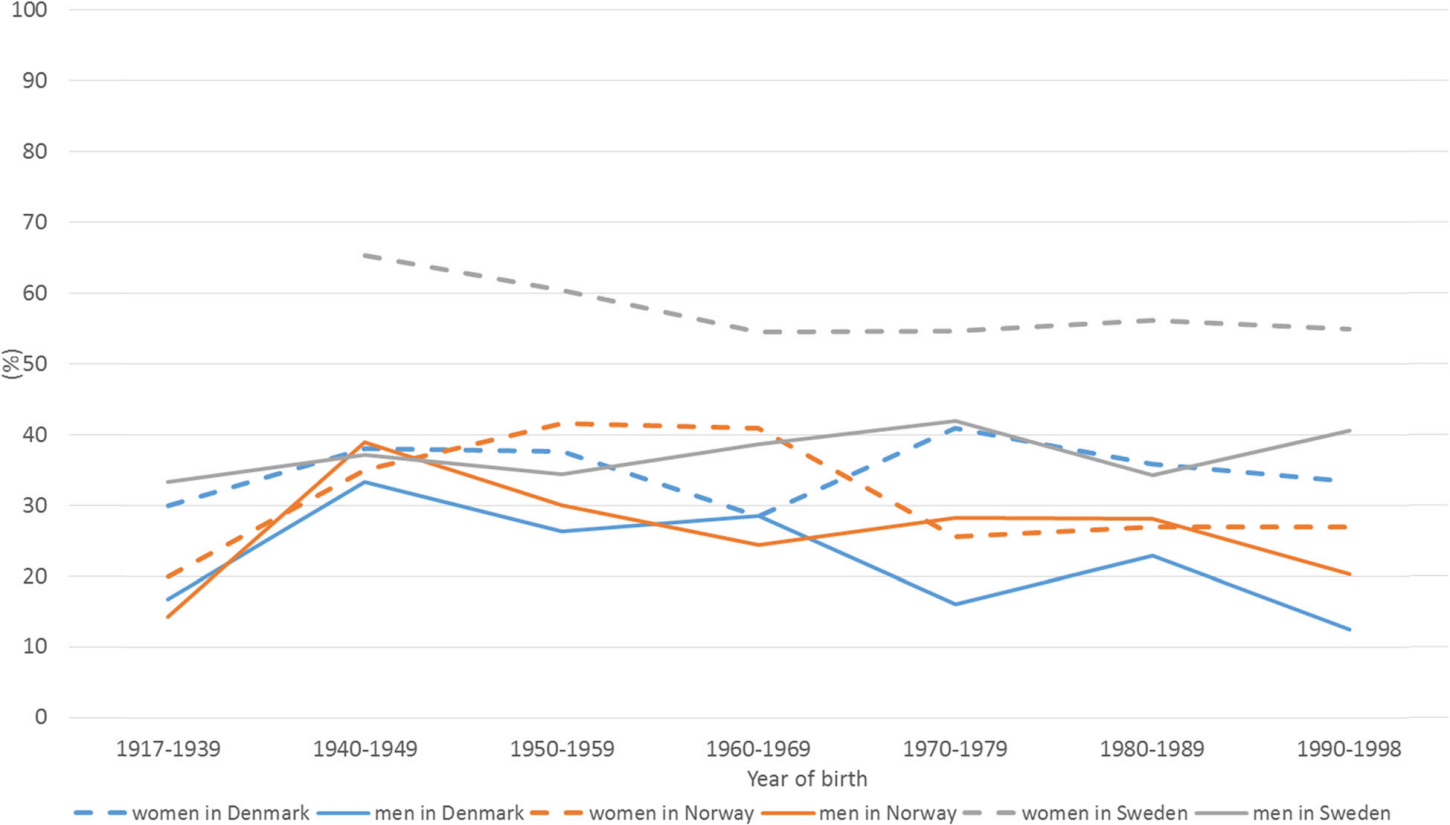
Proportion of respondents who reported that they often or always use three or more protective practices against tick bites, when in areas where there are ticks, by country, gender and decade of birth

**Fig. 5:**
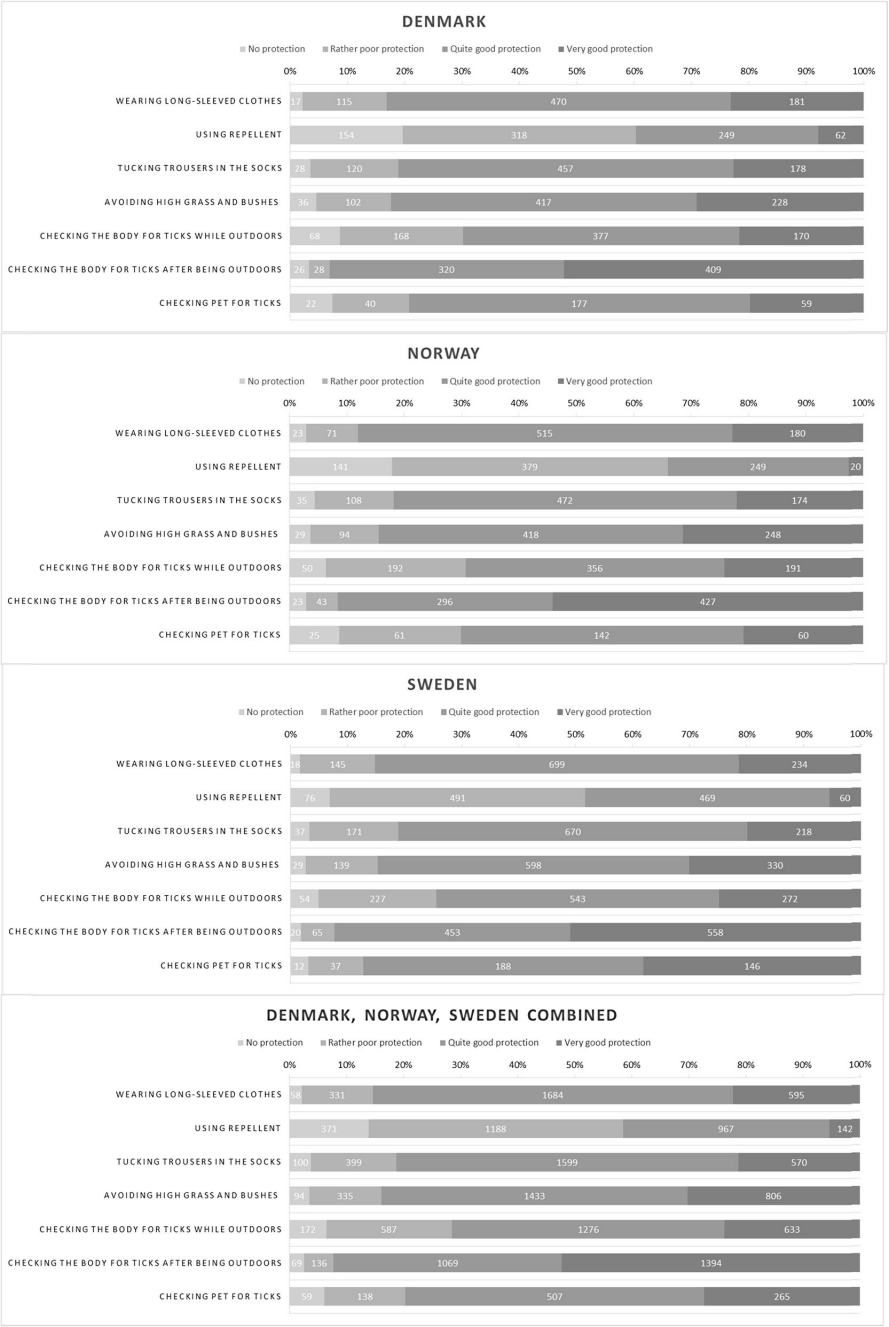
The number and proportion of respondents selecting each answer about perception of the effectiveness of the protective practices against tick bites, by country and the three countries combined

**Fig. 6:**
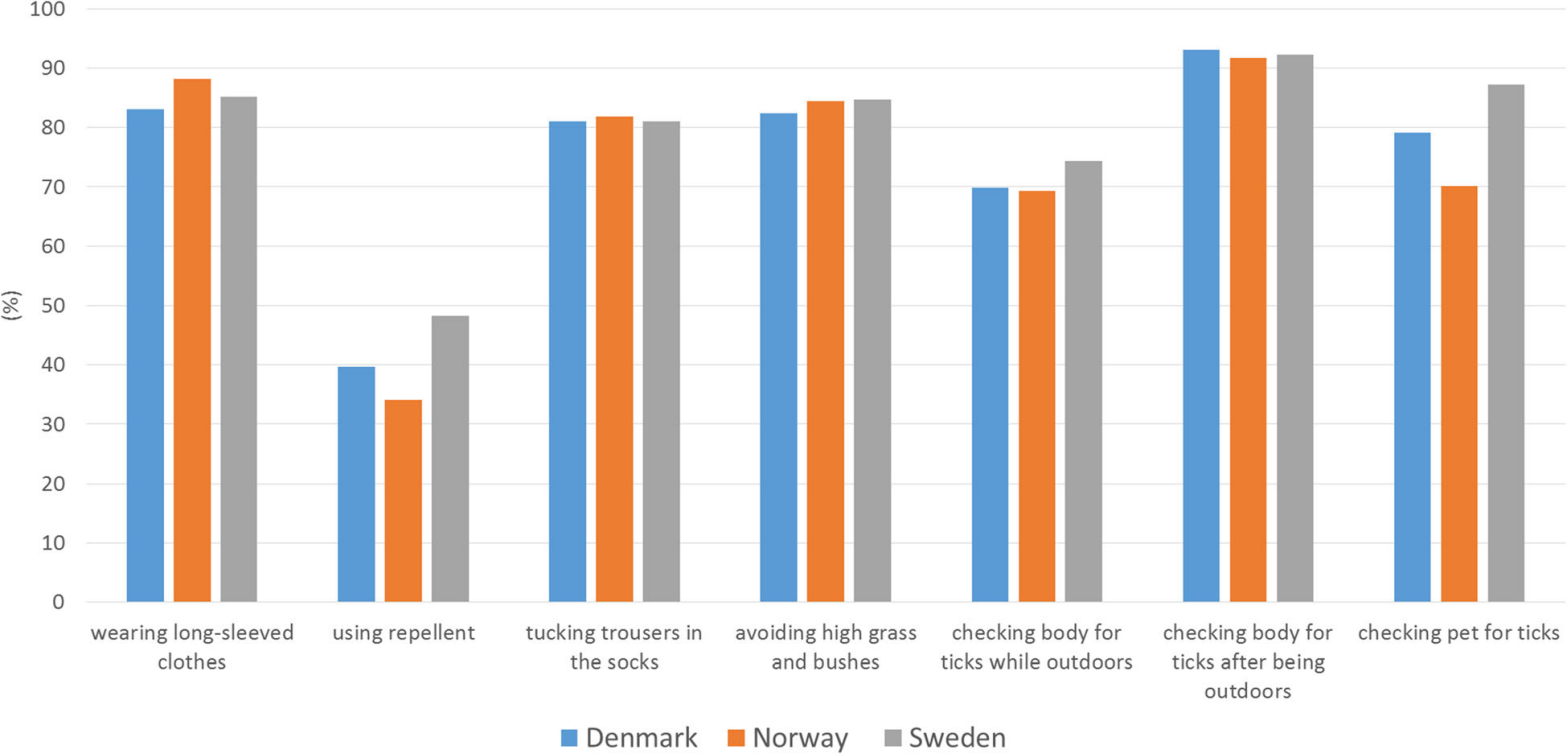
The combined proportion of respondents selecting ‘Quite good protection’ or ‘Very good protection’ for each of the practices, by country. The number of respondents varied by question

We evaluated altogether six explanatory variables in the statistical analyses. Gender was dichotomised (male and others vs. female). Age was initially evaluated as a categorised variable (details not shown) as well as dichotomised at each full 10 years (details not shown); the version of the variable that was selected for the further analyses was dichotomised, at 50 years of age (being under 50 vs. being at least 50 years old). We considered any education after high school or vocational school level as higher education, and the variable included was dichotomised (having higher education vs. not having higher education). It was noted that the youngest respondents had not had time to obtain a higher education. The countries were dichotomised (Denmark or Norway vs. Sweden) based on preliminary results: descriptive results indicated that respondents from Sweden appeared as a separate group from respondents from Denmark and Norway, which appeared to reply similarly, which was supported by visualisation of the data (Figs. 2, 3, 4, 5 and 6) and preliminary logistic regression analyses where the three countries were included as categorised variable (details not shown). Living in the second level of Classification of Territorial Units for Statistics (NUTS 2) regions where the capital cities are located, used as indication of living in urban capital environment, was compared with living in any other NUTS 2 region [14] (living in NUTS 2 region where a capital city is located vs. living in some other NUTS 2 region). As a measure of exposure we included a variable capturing whether the respondents had experience with tick bites. The variable was dichotomised (never been bitten by a tick vs. having experienced one or more tick bites).

First, univariable (crude) logistic regression analyses were performed to evaluate the dichotomous explanatory variables separately for each of the practices. The results were expressed as odds ratios (OR) with 95% confidence intervals (CI), and we considered *P*-values < 0.01 as statistically significant.

Secondly, univariable analyses were performed for using at least three protective practices. Then, a multivariable model for using at least three protective practices was built by first including all six explanatory variables, followed by a backward elimination approach as well as checking for confounding and interaction. We checked for confounding by evaluating whether removal of one variable changed the OR of other variables substantially. We checked for interaction by including the interaction term (variable*variable) and by evaluating whether it was statistically significant. Variables were removed step-wise, starting from the highest *P*-value, until only variables that were significant (P-value < 0.01) and any confounders were kept in the final model. The predictive ability of the model was expressed as the area under the receiver operating characteristic (ROC) curve.

### Ethical statement

The study was approved by the Regional Ethical Review Board at the University of Gothenburg (decision number 714–16). Participants were adults (at least 18 year old) who gave informed consent. Participation was voluntary. Results are reported so that individual participants cannot be identified.

## Results

### Respondents

Of the altogether 2668 respondents, 52.6% were female, 47.0% were male, and 0.4% were of other gender or did not answer the question. Age range was from 18 to 99 years, mean age was 48.6 years. The respondents had an educational level above national averages for higher education in the three countries [15–17]. The descriptive data by country are shown in Table 1.

**Table 1:** Descriptive data of the respondents in the study

	All	Denmark	Norway	Sweden
Mean age [range]	48.6 years [18–99 years]	50.5 years [18–89 years]	47.2 years [18–87 years]	48.2 years [18–99 years]
Proportion of females	52.6%	53.6%	51.5%	52.6%
Proportion not having higher education ^a^	31.3%	22.8%	29.8%	38.4%
Proportion not living in a NUTS 2 where capital city is located	76.8%	68.6%	86.4%	75.8%
Total (n)	2668	783	789	1096

^a^Question about education was only answered by 2616 respondents

The question about education was answered by 2616 respondents. The question about checking pets for ticks was answered by 298, 288 and 383 respondents (total *n* = 969), and the question about checking children for ticks was answered by 226, 258 and 371 respondents (total *n* = 855) from Denmark, Norway and Sweden, respectively. All other variables included had 2668 observations.

### Protective practices

The responses to the questions on the extent of use of protective practices are shown by country in Fig. 2. The two most common protective practices reported to be used often or always against tick bites were wearing long trousers and long-sleeved clothes (53.5%) and checking the body for ticks after being outdoors (57.8%). A majority of the respondents who had pets reported checking them for ticks. Figure 3 shows the combined proportion of respondents who reported ‘Often’ or ‘Always’ using each of the practices by country.

Univariable logistic regression analyses identified several of the six explanatory variables to be associated with using specific protective practices. The odds ratios from univariable logistic regression for each protective practice separately are shown in Table 2. The highest odds ratio detected was 5.29 – the respondents who reported having experienced one or more tick bites had more than five times higher odds to report checking their body after being outdoors than did those respondents who reportedly had experienced no tick bites.

**Table 2:** Odds ratios for each protective practice separately

	Being over 50 years	Being female	Not having higher education ^a^	Being from Sweden	Not living in a NUTS 2 where the capital city is located	Having experienced one or more tick bites
Using long trousers and long-sleeved clothes	1.44 (1.24–1.68)	NS	NS	1.53 (1.31–1.78)	NS	1.37 (1.18–1.60)
Using repellent	NS	1.69 (1.36–2.11)	NS	2.05 (1.65–2.55)	NS	NS
Tucking trousers in socks	1.36 (1.10–1.68)	2.47 (1.96–3.10)	NS	NS	NS	1.43 (1.14–1.78)
Avoiding high grass and bushes	NS	1.71 (1.45–2.00)	NS	1.25 (1.06–1.46)	NS	NS
Checking the body for ticks while outdoors	0.75 (0.63–0.89)	1.38 (1.17–1.63)	NS	2.51 (2.12–2.98)	NS	2.08 (1.74–2.49)
Checking the body for ticks after being outdoors	0.70 (0.60–0.82)	1.66 (1.42–1.93)	0.79 (0.67–0.94)	2.20 (1.87–2.59)	NS	5.29 (4.48–6.26)

Odds ratios (95% confidence interval) are shown for variables that were statistically significantly associated (*P* < 0.01) with each of the protective practices. The variables were dichotomized, each compared with the opposite

*NS* Not significant

^a^These univariable models were based on data from 2616 respondents, all others were based on data from all 2668 respondents

Altogether 1011 respondents (37.9%) reported using at least three different protective practices either often or always when in areas where there are ticks: 522 (19.6%) reported using none, 537 (20.1%) reported using one, 598 (22.4%) reported using two, 512 (19.2%) reported using three, 304 (11.4%) reported using four, 149 (5.6%) reported using five and 46 (1.7%) reported using six protective practices.

Based on univariable models, females had almost two times (1.89, 95% CI 1.58–2.17) higher odds to use at least three protective practices than did respondents of other genders. Respondents from Sweden had two (2.07, 95% CI 1.76–2.43) times higher odds to use at least three protective practices than did respondents from the other two countries. The respondents who reported having experienced one or more tick bites had two (2.09, 95% CI 1.77–2.46) times higher odds to use at least three protective practices than did the respondents who reportedly had experienced no tick bite. In line with these univariable results, based on the multivariable logistic regression model we built, being female, being from Sweden, and having experienced one or more tick bites were positively associated with using at least three different protective practices either often or always when in areas where there are ticks. The odds ratios were 1.90 (95% CI 1.62–2.24), 1.87 (95% CI 1.59–2.21) and 1.88 (95% CI 1.58–2.23), respectively, and the *P*-value for all of three variables was < 0.001. The area under the ROC curve was 0.649, implying the model had moderate predictive power.

Female gender was among the factors identified as positively associated with using at least three different protective practices against tick bites, as well as with several of the practices separately, often or always when in areas where there are ticks. This was particularly pronounced in Sweden (Fig. 4). There was no statistically significant interaction between the variables ‘being female’ and ‘being from Sweden’.

### Perception of protection

Perception of the protection each of the practices provides is shown by country in Fig. 5.

Overall, checking the body for ticks after being outdoors was perceived as offering very good protection by more than half (51.9%) of the respondents, while using repellent was perceived as offering very good protection by a small proportion (5.3%). Figure 6 shows the combined proportion selecting ‘Quite good protection’ or ‘Very good protection’ for each of the practices, by country.

## Discussion

This is the first multi-country study to examine practices against tick bites and thus against tick-borne diseases in Scandinavia. Our study identified interesting patterns in the use of protective practices against tick bites in the study area encompassing three countries. It is generally considered that the Scandinavian countries have similar traditions and behaviour. In this study, we show that this is not the case: when compared with the respondents from Denmark and Norway, the respondents from Sweden had higher odds to use many of the specific protective practices as well as to use at least three protective practices, often or always, when in areas where there are ticks (Table 2, Figs. 2, 3 and 4). The reasons for this difference may include differences in exposure and related risks, awareness, or knowledge; some of these aspects are investigated in other ongoing work based on data from the same questionnaire. Interestingly, similar differences were not evident in relation to the perceived efficacy of different protective measures (Figs. 5 and 6). Hence, country differences in protective practices are most likely not due to differences in perceived efficacy, rather due to other differences, e.g., in exposure.

A major strength of the study was the multi-country approach with many respondents. Compared with other studies of protective practices, our study had a considerably high number of respondents [5, 6, 9]. Furthermore, our selection of respondents aimed for a representative sample of the general population, making the results widely usable. Moreover, our approach was largely descriptive, and the statistical analyses were simple. Main limitations of the study were linked to the questionnaire. The extent of use of the practices was self-reported, and the results may thus be affected by recall bias and reporting bias. Whether the respondents used the practices as they reported remains unconfirmed. It should also be emphasised that the main question analysed in this study did not specify the locations of possible tick encounters – the location could be in another country than the country of residence. For example, Danish residents have acquired tick-borne infections in southern Sweden [18].

As many as 4 out of 5 respondents reported using protective practices against ticks often or always when in areas where there are ticks. However, there is room for improvement: 1 out of 5 respondents reported that they never or rarely used protective practices when in areas where there are ticks. On the other hand, it is encouraging that almost 40% of the respondents used at least three different protective practices often or always when in areas where there are ticks. The most commonly applied practice, checking the body for ticks after being outdoors, was common in all three countries (Figs. 2 and 3) and perceived as being effective, too (Figs. 5 and 6). Wearing long-sleeved clothes was also common (Figs. 2 and 3). These results are in line with the conclusions from an earlier questionnaire-based survey from Sweden that identified using long trousers and long-sleeved clothes and performing tick checks as the most common protective practices [12]. Although the European Centre for Disease Prevention and Control (ECDC) recommends using repellent and clothing against ticks [19], in our study it was seen that a small proportion of respondents reported using repellent or considered it efficient. Similarly, other studies have shown that the use of repellent is the least commonly reported protective practice [8, 10, 12].

The odds to use long trousers and long-sleeved clothes as well as the odds to tuck trousers in socks were higher, whereas checking the body for ticks while outdoors as well as after being outdoors were lower among those older than 50 years than among those who were up to 50 years old (Table 2). Possible reasons for these differences can include practical motives as well as style-reasons: for example, older individuals may need glasses to see ticks, and younger generations may wish to dress differently, e.g., expose their ankles. For checking the body for ticks after being outdoors, previous exposure to one or more tick bites appeared as an important positively associated factor (Table 2). This practice could thus be encouraged particularly among those who have not experienced a tick bite.

One of the most interesting findings of our study was that female gender was among the factors identified as positively associated with using at least three different protective practices against tick bites often or always when in areas where there are ticks. This was particularly pronounced in Sweden (Fig. 4). Gender differences in infection control perception and health behaviour have been described also previously [20, 21]. Our results indicate that it would be useful to target men when planning public information campaigns about protective practices against tick bites in Denmark, Norway and Sweden. The results encourage campaigns to address all age groups, with particular focus on men and the younger generations as well as the elderly (Fig. 4). However, it should be emphasised that an in-depth analysis of why female respondents, especially in Sweden, appeared different in their behaviour (Fig. 4) was beyond the scope of this descriptive study. If intrinsic cultural or socio-economic behavioural traits play a role, the gap may be difficult to bridge by information dissemination strategies.

Tick-borne infections are of emerging and increasing concern in Denmark, Norway and Sweden [1, 2]. A substantial percentage of inhabitants of these three countries are already exposed to the risk of *Ixodes ricinus* bites, and further expansion is expected [22, 23]. Protective practices should be encouraged, and the results of this study can help to motivate and target information campaigns.

## Conclusions

The study sought to identify how people in the three countries protect themselves against tick bites and what they consider effective. Respondent characteristics that were found to be positively associated with protective practices were being female, being from Sweden, and having experienced one or more tick bites. Importantly, the results indicated that men had lower odds to protect themselves against tick bites often or always, when in areas where there are ticks, than women did. To better prevent tick-borne diseases in the general population, men across all ages should be considered a specific target group for information campaigns of relevant authorities.

## Supplementary information

**Additional file 1.** Questionnaire, English version.

## Data Availability

The datasets used and/or analysed during the current study are available from the corresponding author on reasonable request. The questionnaire in the three languages is available upon request.

## Abbreviations

CI: Confidence interval
OR: Odds ratios
